# Mining of two novel aldehyde dehydrogenases (DHY-SC-VUT5 and DHY-G-VUT7) from metagenome of hydrocarbon contaminated soils

**DOI:** 10.1186/s12896-021-00677-8

**Published:** 2021-03-01

**Authors:** Cindy Baburam, Naser Aliye Feto

**Affiliations:** OMICS Research Group, Department of Biotechnology, Faculty of Applied and Computer Sciences, Vaal University of Technology, Vanderbijlpark 1911, South Africa

**Keywords:** Microorganisms, Metagenomics, Aldehyde dehydrogenase, Hydrocarbons, Alkanal, Fosmid clones, Expression host, Bioremediation

## Abstract

**Background:**

Aldehyde dehydrogenases are vital for aerobic hydrocarbon degradation and is involved in the last step of catalysing the oxidation of aldehydes to carboxylic acids. With the global increase in hydrocarbon pollution of different environments, these enzymes have the potential to be used in enzymatic bioremediation applications.

**Results:**

Fifteen fosmid clones with hydrocarbon degrading potential were functionally screened to identify dehydrogenase enzymes. Accordingly, the fosmid insert of the positive clones were sequenced using PacBio next generation sequencing platform and de novo assembled using CLC Genomic Work Bench. The 1233 bp long open reading frame (ORF) for DHY-SC-VUT5 was found to share a protein sequence similarity of 97.7% to short-chain dehydrogenase from *E. coli.* The 1470 bp long ORF for DHY-G-VUT7 was found to share a protein sequence similarity of 23.9% to glycine dehydrogenase (decarboxylating) (EC 1.4.4.2) from *Caulobacter vibrioides* (strain NA1000 / CB15N) (*Caulobacter crescentus*). The in silico analyses and blast against UNIPROT protein database with the stated similarity show that the two dehydrogenases are novel. Biochemical characterization revealed, that the highest relative activity was observed at substrate concentrations of 150 mM and 50 mM for DHY-SC-VUT5 and DHY-G-VUT7, respectively. The K_m_ values were found to be 13.77 mM with a V_max_ of 0.009135 μmol.min^− 1^ and 2.832 mM with a V_max_ of 0.005886 μmol.min^− 1^ for DHY-SC-VUT5 and DHY-G-VUT7, respectively. Thus, a potent and efficient enzyme for alkyl aldehyde conversion to carboxylic acid.

**Conclusion:**

The microorganisms overexpressing the novel aldehyde dehydrogenases could be used to make up microbial cocktails for biodegradation of alkanes. Moreover, since the discovered enzymes are novel it would be interesting to solve their structures by crystallography and explore the downstream applications.

**Supplementary Information:**

The online version contains supplementary material available at 10.1186/s12896-021-00677-8.

## Background

Anthropogenic disturbances such as hydrocarbon contamination in soil environments can affect the microbial community structure and diversity since it is toxic to microbial cells [1, 2]. Some microorganisms have the ability to use hydrocarbons as a sole carbon source thus, such soils represent a complex environment teeming with microbial diversity, which is preferred for gene mining and natural product discovery [3]. Hydrocarbon-polluted soil sites can be targeted with confidence in identifying novel aldehyde dehydrogenases (ALDHs) involved in aerobic pathways for petroleum hydrocarbon breakdown [4]. Furthermore, metagenomic DNA from these soils reveal the potential of unculturable microorganisms that are otherwise troublesome to culturing techniques as a source of novel enzymes [5]. Numerous studies have shown the success of employing this technique for example novel lipases [6], alkane hydroxylases [7], monooxygenases [8, 9], alcohol dehydrogenases [10] to list but a few. Thus, enzymes are sought-after biomolecules for their potential in industrial processes and environmental bioremediation [11].

The ALDHs are a diverse group of related enzymes that catalyze the oxidization of aldehydes to carboxylic acids, using NAD^+^ or NADP^+^ as the coenzyme. ALDHs are ubiquitous in all life forms and are classified as detoxification enzymes involved with coping with toxic aldehydes generated from various cellular metabolisms. However, more specifically related to this research, aldehyde dehydrogenases are involved in numerous bioconversion processes for example the degradation of alkanes in which alkyl aldehydes are generated as intermediates and represents the third step of the alkane degradation pathway. Due to their specific function, these biocatalysts are of great interest for industrial applications [12].

To date there are only a few reports of ALDHs of bacterial origin involved in oxidation of short and medium chain alkyl aldehydes (C_1_ – C_14_) such as those from *Acinetobacter* sp. strain M-1 (Ald1, C_4_–C_14_) [13], *Geobacillus thermodenitrificans* NG80–2 (C_1_ – C_20_) [14], *Rhodococcus erythropolis* (ThcA, C_1_–C_8_), *Acetobacter pasteurianus* SKU1108 [15] and *Alcanivorax borkumensis* SK2T [16]. Recently, a novel bifunctional aldehyde/alcohol dehydrogenase was identified for effective ethanol fermentation pathway in hyperthemophiles thus enabling cellulosic ethanol production [17]. Other research has also shown a similar co-expression of alcohol dehydrogenase and aldehyde dehydrogenase in *Bacillus subtilis* for alcohol detoxification [18].

Petroleum hydrocarbons are one of the most difficult-to-degrade environmental pollutants that continue to pose serious challenges to human, animal, plant and inhabitants of marine and soil environments. Biodegradation or bioconversion of such pollutants to less harmful products necessitates the involvement of a set of enzymes at different level of the catabolic reactions. To the effect, aldehyde dehydrogenases (ALDHs) are known to be involved in the aerobic pathways of petroleum hydrocarbon breakdown. Several findings have been reported regarding sourcing of ALDHs from plant, animal, as well as some from microbial origins using culture-based method. Here we report the discovery of two novel ALDHs sourced from metagenome of hydrocarbon-contaminated soil. We believe the discovered novel ALDHs would perfectly fit in the hydrocarbon degradation pathways and eventually become part of the enzyme cocktail that could be engineered for petroleum hydrocarbon degradation, pending further studies.

## Results

### The quality of isolated fosmid DNA

Fosmid DNA was isolated from 15 fosmid clones selected on the basis of a functional screening procedure. This was based on their ability to utilize one of the three hydrocarbon substrates i.e. hexadecane, cyclohexane and octadecene. The isolated fosmid DNA was separated using agarose gel electrophoresis and was found to be of high quality and quantity. This was important for downstream PACBIO SMRT® next generation sequencing that requires high quality and quantity DNA to ensure successful long sequence reads.

### Sequence analysis and ALDH gene synthesis

De novo assembly was carried out on the PACBIO sequences for the 15 fosmid clones using the CLC Genomics Workbench 11.0.1 (CLC Bio, Qiagen). The longest assembled contigs for each clone obtained from this software program was used to obtain open reading frames and consequently find regions of local similarity between known sequences to identify aldehyde dehydrogenase genes involved in the third step of aerobic hydrocarbon degradation Uniprot-Swiss prot (https://www.uniprot.org/) database. An ORF of 1233 bp in length encoding a polypeptide of 406 amino acids with 50.6 kDa predicted molecular mass was compared to UniProt protein database using BlastP and found to share a protein sequence similarity of 97.7% to short-chain dehydrogenase from *E. coli.* The complete *dhy-sc-vut5* nucleotide sequence was submitted to GenBank and assigned an accession number MT606180.

The second ORF of 1470 bp encoding a polypeptide of 637 amino acids with a predicted molecular mass of 67.6 kDa was found to share a protein sequence similarity of 23.9% to glycine dehydrogenase (EC 1.4.4.2) from *Caulobacter vibrioides* (strain NA1000 / CB15N) (*Caulobacter crescentus*). The complete *dhy-g-vut7* nucleotide sequence was submitted to GenBank and assigned an accession number MT606181.

### Cloning, expression and protein purification

The extracted plasmid DNA was digested with *Xba*I and *Hind*III to confirm the ligation reaction. This was found to be of high quality and the restriction digests showed the successful cloning of the insert DNA with the expected band sizes of 1233 bp (pET30(+)_dhy-sc-vut5) and 1470 bp (pET30(+)_dhy-g-vut7). The expression of high levels of recombinant proteins is essential for this study hence the two modified gene were cloned into a pET30a (+) expression vector.

**Fig. 1:**
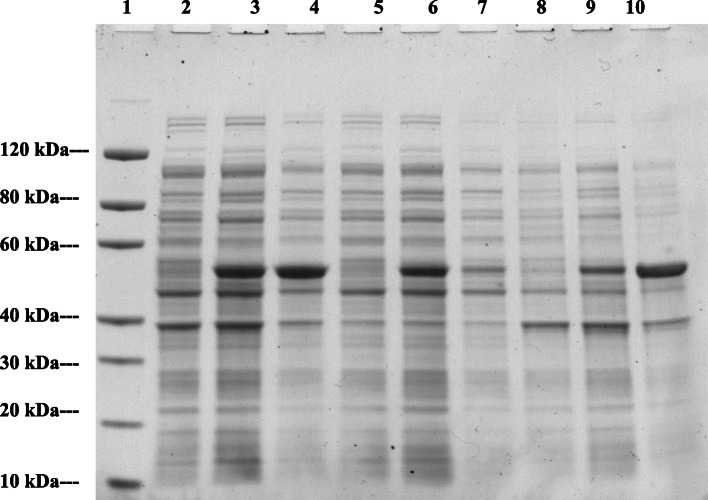
SDS PAGE analysis of soluble and insoluble fractions of the cell extracts of DHY-G-VUT7 to determine the protein profile at varying conditions of induction. Lane 1: Protein Marker, GenScript, Lane 2: Cell lysate without induction, Lane 3: Cell lysate with induction for 16 h at 15 °C, Lane 4: Cell lysate with induction for 4 h at 37 °C, Lane 5: Supernatant of cell lysate without induction, Lane 6: Supernatant of cell lysate with induction for 16 h at 15 °C, Lane: 7 Supernatant of cell lysate with induction for 4 h at 37 °C, Lane:8 Pellet of cell lysate without induction, Lane 9: Pellet of cell lysate with induction for 16 h at 15 °C and Lane: 10 Pellet of cell lysate with induction for 4 h at 37 °C

**Fig. 2:**
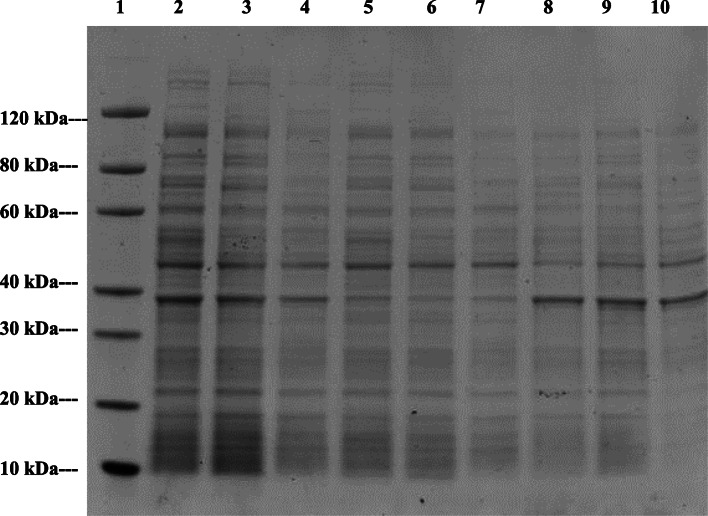
SDS PAGE analysis of soluble and insoluble fractions of the cell extracts of DHY-SC-VUT5 to determine the protein profile at varying conditions of induction. Lane 1: Protein Marker, GenScript, Lane 2: Cell lysate without induction, Lane 3: Cell lysate with induction for 16 h at 15 °C, Lane 4: Cell lysate with induction for 4 h at 37 °C, Lane 5: Supernatant of cell lysate without induction, Lane 6: Supernatant of cell lysate with induction for 16 h at 15 °C, Lane: 7 Supernatant of cell lysate with induction for 4 h at 37 °C, Lane:8 Pellet of cell lysate without induction, Lane 9: Pellet of cell lysate with induction for 16 h at 15 °C and Lane: 10 Pellet of cell lysate with induction for 4 h at 37 °C

The soluble and insoluble fractions of the cell extracts of the dehydrogenase proteins were analysed on SDS-PAGE to determine the expression level of the proteins and the protein profile following varying conditions of induction (Figs. 1 and 2).

**Fig. 3:**
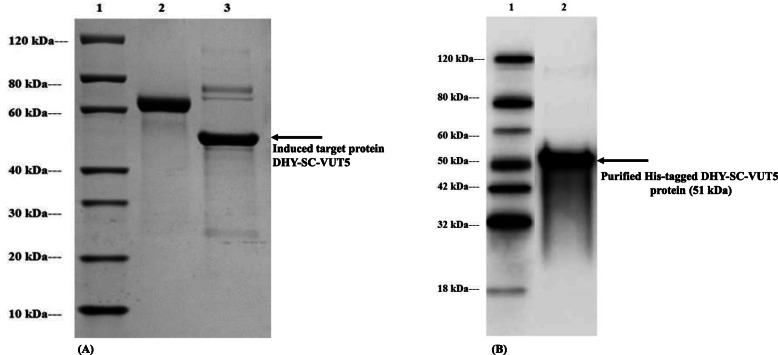
**a** SDS PAGE analysis of novel aldehyde dehydrogenase (DHY-SC-VUT5) with the protein band corresponding to a size of 51 kDa after overexpression in BL21 (DE3) *E. coli* cells following induction at 15 °C for 16 h with 0.5 mM IPTG and purification using the Ni column. Lane 1: Protein Marker, GenScript, Lane 2: BSA (2 μg) and Lane 3: Overexpressed purified DHY-SC-VUT5 protein (2 μg). **b** Western blot analysis of DHY-SC-VUT5 protein probed with anti-His antibody. Lane 1: Protein Marker, GenScript, Lane 2: purified His-tagged DHY-SC-VUT5 protein of approximately 51 kDa. DHY-SC-VUT5 contains a C-terminal 6xHis tag and was purified using Ni-nitrilotriacetic acid (Ni-NTA) column

**Fig. 4:**
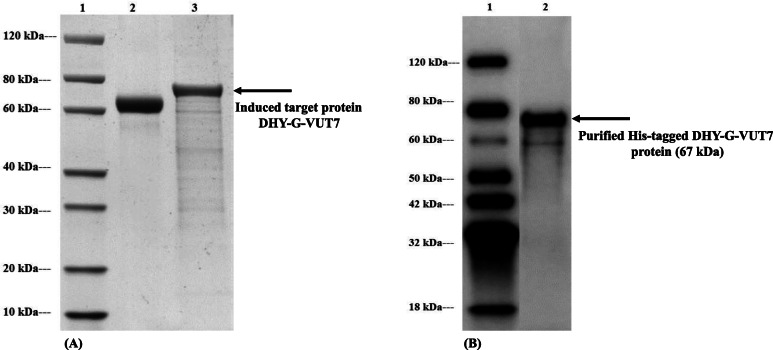
**a** SDS PAGE analysis of novel aldehyde dehydrogenase (DHY-G-VUT7) with the protein band corresponding to a size of 67 kDa after overexpression in BL21 (DE3) *E. coli* cells following induction at 15 °C for 16 h with 0.5 mM IPTG and purification using the Ni column. Lane 1: Protein Marker, GenScript, Lane 2: BSA (2 μg) and Lane 3: Overexpressed purified DHY-G-VUT7 protein (2 μg). **b** Western blot analysis of DHY-G-VUT7 protein probed with anti-His antibody. Lane 1: Protein Marker, GenScript, Lane 2: purified His-tagged DHY-G-VUT7 protein of approximately 67 kDa. DHY-G-VUT7 contains a C-terminal 6xHis tag and was purified using Ni-nitrilotriacetic acid (Ni-NTA) column

The estimated molecular mass of the individual proteins was calculated from the respective amino acid sequences to compare to the standard protein marker. This was found to be 50.6 kDa and 67.6 kDa for DHY-SC-VUT5 and DHY-G-VUT7 respectively. Varying experimental conditions for induction such as time and temperature were setup to maximize recombinant protein expression levels. The images of SDS PAGE gels for induced short-chain dehydrogenase (DHY-SC-VUT5) and glycine dehydrogenase (DHY-G-VUT7) proteins expressed in BL21 (DE3) upon purification are represented in Fig. 3(a) and Fig. 4(a) respectively. The results showed high quality and successful protein induction and expression.

The purified proteins exhibited a band of ~ 67 kDa and ~ 51 kDa for the induced DHY-G-VUT7 and DHY-SC-VUT5 proteins, respectively from the cell lysate. The bands were confirmation that both proteins were successfully overexpressed, purified and corresponded to the calculated molecular mass. The optimal induction conditions were achieved at 15 °C upon incubation period of 16 h for both proteins using 0.5 mM IPTG. The protein concentrations were achieved using the Bradford protein assay which revealed 0.62 mg/ml for DHY-SC-VUT5 and 0.53 mg/ml for DHY-G-VUT7 respectively.

Western blot analysis was carried out to confirm the purity, molecular weight and expression of the target proteins observed on the SDS PAGE gel. The images of Western blot analysis [Fig. 3(B) and Fig. 4(B)] showed the binding of the anti-His antibody to both short-chain dehydrogenase band (DHY-SC-VUT5) and glycine dehydrogenase band (DHY-G-VUT7) containing 6x His-tags with an expected band size of 51 kDa and 67 kDa, respectively. Thus, confirming the successful expression and purification of both target proteins.

### Optimal aldehyde dehydrogenase (DHY-SC-VUT5 and DHY-G-VUT7) concentration

Varying concentrations of the purified DHY-SC-VUT5 and DHY-G-VUT7 enzymes (0.001 mg/ml, 0.003 mg/ml, 0.007 mg/ml and 0.014 mg/ml) were tested individually using the ADH assay as stipulated by Li et al. [14]. This was carried out to determine the optimal concentration of enzyme to be used for the kinetics study and cocktail of enzymes. The effect of varying enzyme concentrations in the assay was achieved by change in absorbance value at a wavelength of 340 nm indicative of the amount of NADH formed by the reduction of the coenzyme NAD^+^ for individual enzyme concentrations of DHY-SC-VUT5 and DHY-G-VUT7. Absorbance data and graphs for the assays shown in supplementary data.

**Fig. 5:**
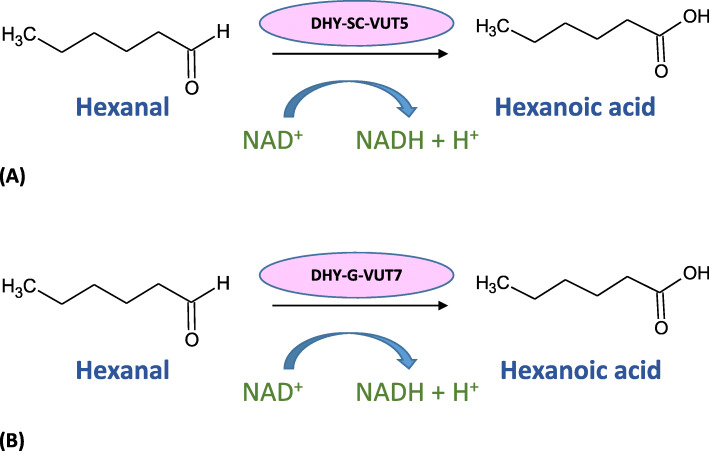
Schematic representation of the enzymatic reaction of the two identified novel aldehdyde deydrogenase DHY-SC-VUT5 (**a**) and aldehdyde dehydrogenase DHY-G-VUT7 (**b**)

The gradual amount of NAD^+^ reduced to NADH was recorded over the time interval, indicated by an increase in absorbance values**.** The optimal enzyme concentration for DHY-SC-VUT5 and DHY-G-VUT7 were observed to be 0.003 mg/ml. DHY-SC-VUT5 displayed higher NADH production in comparison to DHY-G-VUT7 with an absorbance value of 0.207 a.u and 0.131 a.u respectively. Thus, the concentration was used for subsequent enzymatic kinetic studies. Maximum NADH production took place within the first 120 s suggesting that the reaction rate was very fast. This showed the reduction of NAD^+^ to NADH exhibits strong UV absorption at 340 nm and an increase in absorbance value is indicative of the amount of NADH produced when catalysed by the enzyme [14]. From the current study, it can be concluded that the class of dehydrogenase enzyme identified for DHY-SC-VUT5 and DHY-G-VUT7 proteins are indeed novel aldehyde dehydrogenases with the ability to convert hexanal to hexanoic acid shown by the reduction of coenzyme NAD^+^ to NADH. This reaction has been schematically represented in Fig. 5 for both proteins.

The results showed that an increase in enzyme concentration has a direct effect on the amount of NAD^+^ converted to NADH as indicated by an increase in the relative activity. The two-way analysis of variance (ANOVA) showed that the concentration of DHY-SC-VUT5 and DHY-G-VUT 7 novel enzymes accounted for 42.88% (*p* < 0.0001) and 22.3% (*p* = 0.0003) of the total variance, respectively. Thus, the effect of enzyme concentration on NADH production can be considered very significant.

### Optimal hexanal substrate concentration and enzyme kinetics

Varying concentrations of the substrate (hexanal) were tested in the ALDH assay as stipulated by Li et al. [14]. This was carried out to determine the optimal concentration of substrate to be used for the kinetics study. The effect of varying substrate concentrations in this assay is depicted by change in absorbance value at a wavelength of 340 nm. This is indicative of the amount of NADH formed by the reduction of the coenzyme NAD^+^ hence the direct conversion of hexanal to hexanoic acid. Absorbance data and graphs for the assays shown in supplementary data. The equimolar amount of the two enzymes was added to the reaction mixture for the kinetic and comparative studies of the enzymes.

The two-way analysis of variance (ANOVA) confirmed that the concentration of hexanal affects the relative activity and accounts for 12.29% (*p* = 0.0010) and 22.46% (*p* = 0.0001) total variance for DHY-SC-VUT5 and DHY-G-VUT7, respectively. Thus, the effect of substrate is considered extremely significant to the change in relative activity observed for both enzymes.

**Fig. 6:**
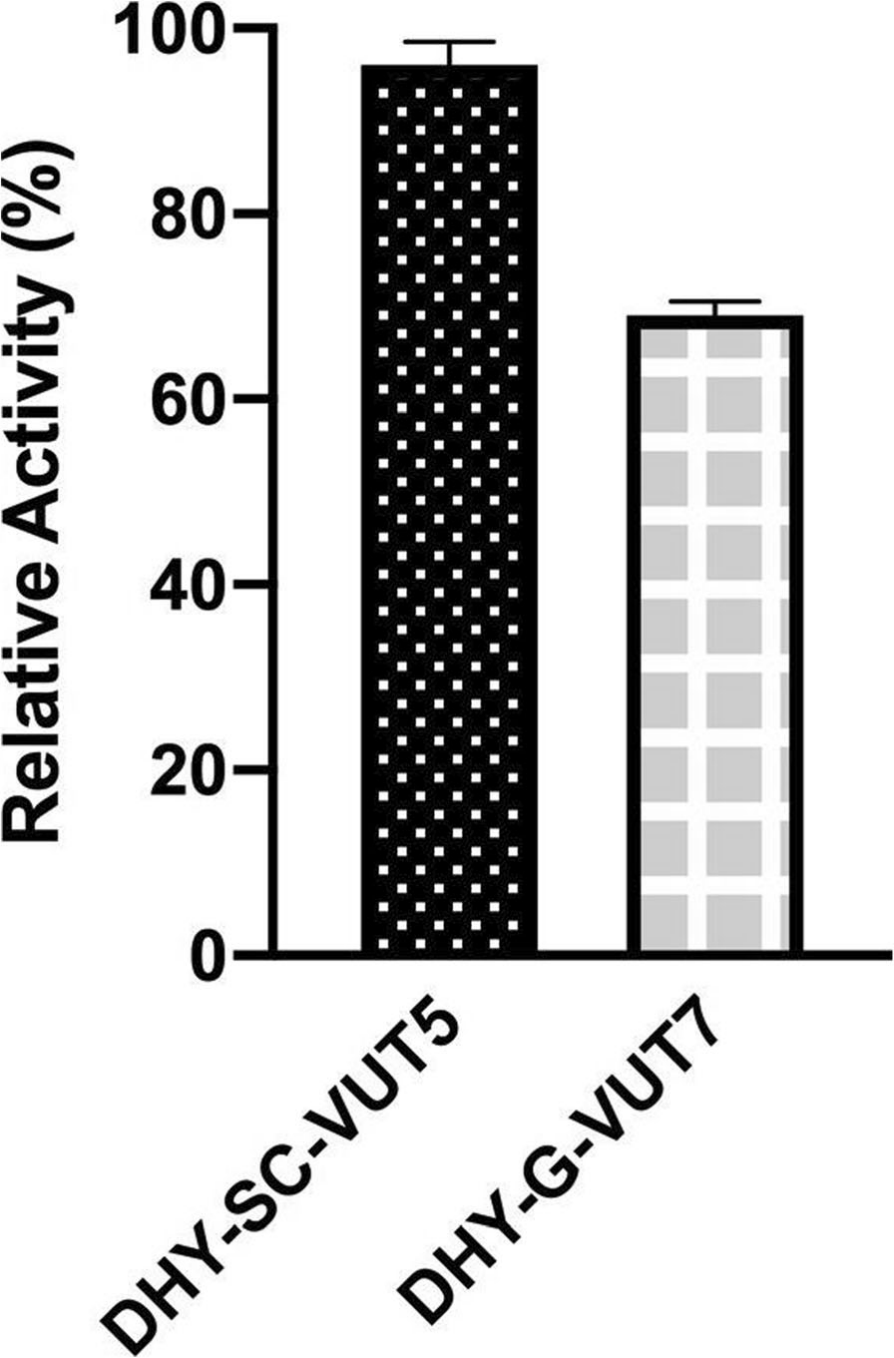
Comparison of the relative activities of two novel aldehyde dehydrogenase DHY-SC-VUT5 and DHY-G-VUT7 using hexanal as a substrate over a time interval of 3 min at 30 °C. The data was analysed using GraphPad Prism Version 8.4.1 (460)

The relative activity of both enzymes was subjected to comparison at equimolar concentration (50 mM) of the two enzymes at a time interval of 3 min using hexanal as a substrate (Fig. 6). The results showed marked difference between the relative activity of DHY-SC-VUT5 and DHY-G-VUT7 with the former exhibiting the highest activity for the same substrate. The difference can be attributed to the relative activity of the individual enzymes which accounted for 28.85% of the total variance and the effect which was considered extremely significant (*p* < 0.0001) as revealed by 2-way ANOVA.

**Fig. 7:**
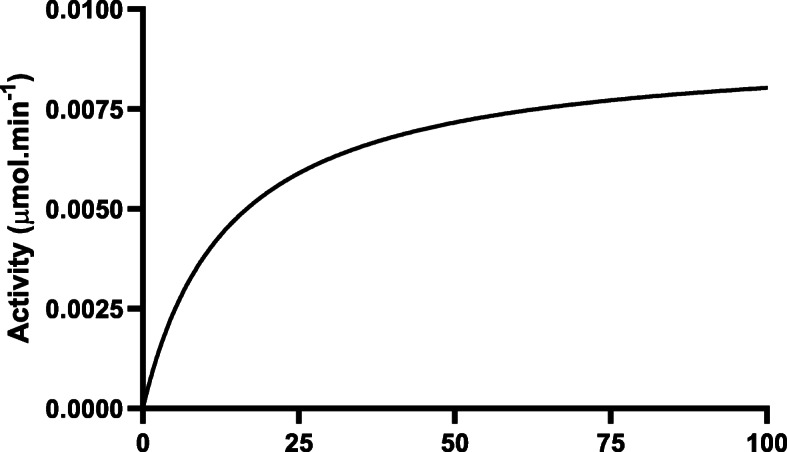
Enzymatic activity of novel aldehyde dehydrogenase DHY-SC-VUT5 at different concentration of hexanal at 30 °C. Absorbance values were recorded spectrophotometrically at 340 nm wavelength. The kinetic data was fitted into Michaelis-Menten model using GraphPad Prism Version 8.4.1 (460)

**Fig. 8:**
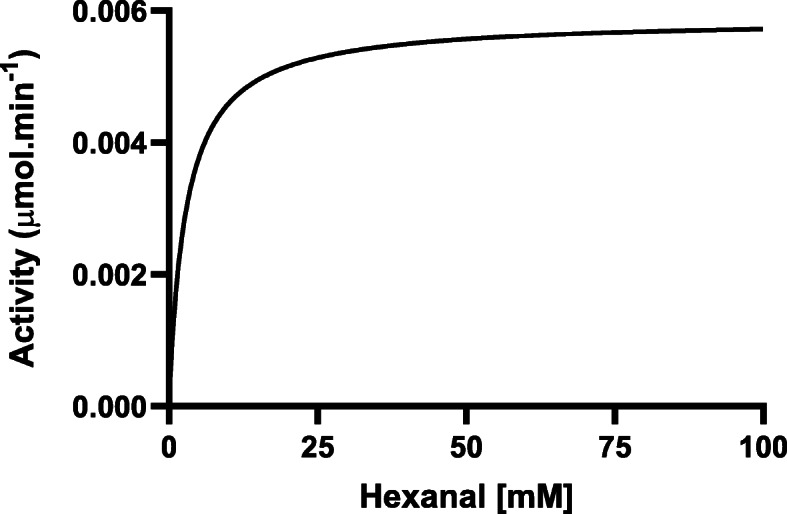
Enzymatic activity of novel aldehyde dehydrogenase DHY-G-VUT7 at different concentration of hexanal at 30 °C. Absorbance values were recorded spectrophotometrically at 340 nm wavelength. The kinetic data was fitted into Michaelis-Menten model using GraphPad Prism Version 8.4.1 (460)

The absorbance values for the varying hexanal substrate concentrations were used to calculate the velocity and to construct the Michaelis-Menten graphs using Graph Pad Prism Version 8.4.1 (460) for DHY-SC-VUT5 (Fig. 7) and DHY-G-VUT7 protein (Fig. 8). The graphs describe the rate of the enzymatic reaction by relating reaction rate (rate of formation of product) to the concentration of a substrate. It can be deduced that the enzymatic reactions of both graphs followed a typical first order for Michaelis-Menten function when hexanal was used as a substrate in the presence of NAD^+^. Thus, the rate of the enzymatic reactions is directly proportional to the concentration of the reacting substance. The kinetics data clearly demonstrates that both enzymes, DHY-SC-VUT5 and DHY-G-VUT7, function as aldehyde dehydrogenases.

The K_m_ value was found to be 13.77 mM with a V_max_ of 0.009135 μmol.min^− 1^ and 2.832 mM with a V_max_ of 0.005886 μmol.min^− 1^ for DHY-SC-VUT5 and DHY-G-VUT7, respectively. The relationship between rate of reaction and concentration of substrate depends on the affinity of the enzyme for its substrate.

## Discussion

### Identification and novelty of isolated genes

The percentage protein sequence similarities of the aldehyde dehydrogenase DHY-SC-VUT5 (97.7%) and glycine dehydrogenase DHY-G-VUT7 (23.9%) to short-chain and glycine dehydrogenases in the protein database, respectively, could be attributed to their novelty. Moreover, the ORF lengths identified are similar to those seen in the literature for other isolated aldehyde dehydrogenases [13, 19]. The dehydrogenase showed sequence similarity to that isolated from *Caulobacter vibrioides* (strain NA1000 / CB15N) (*Caulobacter crescentus*) according to the Uniprot database search. *Caulobacter vibrioides* is from the taxonomic lineage *Proteobacteria* which was shown to display an 84% phylum abundance in the soil samples according to microbiome profiling studies carried out in a previous study. Work carried out by Yang et al. [20], which involved identifying potential degraders within bacterial profiles, showed that oil samples were dominated by the bacterial taxa of *Caulobacteraceae* (esp. *Caulobacter*). Their study showed that they were either tolerant to or capable of degrading crude oil compounds especially, PAHs. The findings have also been supported by previous studies [21, 22]*.*

### Enzymatic activity

Since a small K_m_ value indicates that the enzyme has a high affinity for the substrate, it can be concluded that both enzymes displayed a high affinity for hexanal as a substrate. By using 13.77 mM (DHY-SC-VUT5) and 2.832 mM (DHY-G-VUT7) hexanal, the proteins reached half of V_max_ and thus, acts at a more or less constant rate, regardless of variations in the concentration of substrate within the physiological range. However, DHY-G-VUT7 importantly shows a higher affinity for hexanal than DHY-SC-VUT5 due to its much lower K_m_ value thus making DHY-G-VUT7 a more potent and efficient enzyme for alkyl aldehyde degradation. The K_m_ values obtained for both aldehyde dehydrogenases are similar to a membrane bound aldehyde dehydrogenase complex AldFGH involved in acetic acid fermentation isolated from *Acetobacter pasteurianus* SKU1108 [15].

### Application of aldehyde dehydrogenases

It is important to note that the novel dehydrogenase genes identified here have the potential to convert the harmful compound hexanal into a less toxic compound, hexanoic acid. Moreover, the fosmid clones were selected based on functional screening on hydrocarbon substrates thus, the increased probability of the dehydrogenase involvement in the hydrocarbon degradation pathway specifically alkyl aldehydes. There are a number of studies that have implemented the metagenomics approach similar to the one in this study [22–25]. Some industrially relevant enzymes discovered by metagenomics include cellulases [26], amylases [27], chitinases [28], lipases [29], carboxylesterase [30], alcohol dehydrogenase [31], aldehyde dehydrogenase [19] and proteases [32].

Hexanal is an intermediate in the transformation of toxic hexanol to the non-toxic carboxylic acid [33]. This intermediate reaction would not be possible without aldehyde dehydrogenases [34]. Accumulation of hexanal in the soil as a result of the slow transformation process by indigenous microorganisms is a contributing factor in soil pollution [35]. Due to this slow process and the toxic nature of this compound, there is a need to develop an enzyme-based solution to speed up the degradation process by identifying novel aldehyde dehydrogenases as successfully done in this study. Furthermore, the resulting carboxylic acids are beneficial for promoting soil fertility and plant growth [36] besides its recorded industrial application.

There have been aldehyde dehydrogenases identified and purified for application in hydrocarbon break down. For instance, Ishige et al. (200) identified aldehyde dehydrogenase (55,496 Da) *Acinetobacter* sp. strain M-1 with activity against *n*-alkanals (C4 to C14), with a preference for longer carbon [13]. The strain M-1 was able to utilize hexadecane as a sole carbon source because of its ability to produce aldehyde dehydrogenase. Moreover, a bi-functional 3-hydroxybutanal dehydrogenase or reductase sourced from *Desulfococcus biacutus* involved in acetone metabolism was cloned and functionally expressed [24, 37]. The enzyme was reported to show the highest activities in reduction of C3 - C5-aldehydes with NAD^+^.

Another study identified for the first time a bacterial aromatic aldehyde dehydrogenase (ALDH) obtained from 20 putative ALDH genes of *Sphingobium* sp. strain SYK-6 critical for the efficient catabolism of syringaldehyde. Syringaldehyde is obtained from lignin and are essential intermediates for the production of basic chemicals using microbial cell factories [38]. Furthermore, a study showed the elevated expression of ALDH in earthworms in heavy metal polluted soils [39]. Hence, this was used as a bioindicator to monitor heavy metal pollution in soil. The ALDH activity was found to be highest in worms cultured in 5 ppm heavy metal contaminated soils. Their study showed an accumulation in earthworms in these soils since they ensure that the toxic aldehydes are neutralized to carboxylic acids as a stress responsive method. All these studies further highlight the importance of our newly discovered aldehyde dehydrogenases for application in detoxifying various pollutants.

## Conclusion

In conclusion, we have discovered two novel dehydrogenases (DHY-SC-VUT5 and DHY-G-VUT7) from the metagenome of hydrocarbon contaminated soils. The DHY-G-VUT7 dehydrogenase showed higher substrate affinity than DHY-G-VUT7. The high affinity of both enzymes for hexanal as a substrate is valuable for their application in the breakdown of alkyl aldehyde compounds that persist in the soil and water environments. Despite being promising candidates to play part in the hydrocarbon degradation, the two novel enzymes have a bottleneck, which is the need to apply the expensive cofactor NAD^+^ for the enzymes to work. Interestingly, there are microorganisms that naturally or metabolically engineered to bio-generate NAD^+^. Such microorganisms could potentially play key role in continuously generating the cofactor that could be used by the enzymes. Therefore, a microbial cocktail could be engineered from a mixture of microorganisms overexpressing the novel genes and those producing other enzymes that play role at different stages of hydrocarbon degradation among which aldehyde dehydrogenase is one and microorganisms that generate NAD^+^. Thus, microbial cocktail that can be engineered could potentially be applied for bioremediation of hydrocarbon pollution in both soil and water environments. However, further research should be conducted in order to establish the synergy, compatibility and the stability of the to-be-developed microbial cocktail.

## Methods

### Selection of fosmid clones

A total of 15 candidate fosmid clones from the prepared metagenomic library were selected based on functional screening analysis using three hydrocarbon substrates (hexadecane, octadecene and cyclohexane). Five clones were chosen for each of the substrates based on their growth rate and colony size on minimal media (Bushnell Haas media) supplemented with a hydrocarbon source (Table 1).

**Table 1:** Selected fosmid clones based on growth rates on three different hydrocarbon substrates

Hydrocarbon substrates	Selected clones
Hexadecane	pFos_A1, pFos_A7, pFos_B2, pFos_B3 and pFos_F2
Octadecene	pFos_D3, pFos_D8, pFos_F1, pFos_F3 and pFos_A4
Cyclohexane	pFos_A2, pFos_C7, pFos_B7, pFos_D7 and pFos_G3

### Induction of fosmid clones and extraction of fosmid DNA

An overnight culture of each fosmid clone was grown in 10 ml of Luria-Bertani (LB) broth supplemented with chloramphenicol (12.5 μg/ml) to make a stock culture and 1 ml was used to inoculate 25 ml LB broth supplemented with chloramphenicol (12.5 μg/ml). This was autoinduced by adding 50 μl 500X CopyControl™ Fosmid Autoinduction Solution as previously described by the manufacturer (Epicentre, USA). The autoinduced culture was grown overnight at 37 °C in a shaking incubator at 170 rpm. EPI 300™ bacterial host cells grown in 25 ml LB broth was used as the negative control. The bacterial cells were harvested from 10 mls of overnight culture by centrifugation at 4000 rpm for 5 min. The supernatant was discarded, and the pellet resuspended in 250 μl suspension buffer (Epicentre, USA). The fosmid DNA was, thereafter, extracted using the GeneJET Plasmid Miniprep Kit (Thermo Fisher Scientific, Waltham, USA) according to the manufacturer’s instructions. Isolated fosmid DNA was eluted in 50 μl Elution buffer supplied by the manufacturer and stored at − 80 °C.

### Fosmid DNA construct integrity assessment

The extracted fosmid constructs from 15 positive fosmid clones were visualised on a 0.8% (w/v) agarose gel stained with ethidium bromide and run at 100 V for 30 min (data not shown, can be supplied if requested). The pCC2FOS™ vector supplied with the CopyControl™ HTP Fosmid Library Production Kit (Epicentre, USA) was run on the agarose gel electrophoresis as a control. The fosmid constructs were then sequenced using PACBIO SMRT® next generation sequencing platform at Inqaba Biotec™ (Pretoria, South Africa).

### De novo sequence assembly and analysis

De novo assembly was carried out on the PACBIO sequences obtained from Inqaba Biotec™ using the CLC Genomics Workbench 11.0.1 (CLC Bio, Qiagen). The quality of data was checked, the contig sequence length was set at 200 bp and filtering was carried out based on default parameters in the software. The software program was used to extract the longest contig assembly. The open reading frames along with the translated protein were determined from the assembled contig sequences for each of the fifteen fosmid clones using ORF finder on the NCBI database (https://www.ncbi.nlm.nih.gov/orffinder/). A protein Basic Local Alignment Search Tool (BLASTp) called Uniprot-Swiss prot (https://www.uniprot.org/) was carried out on the individual ORF sequences in order to find regions of local similarity between known sequences to identify members of gene families involved in hydrocarbon degradation pathways. These ORFs were flagged for further analysis.

### Gene synthesis and cloning

**Fig. 9:**
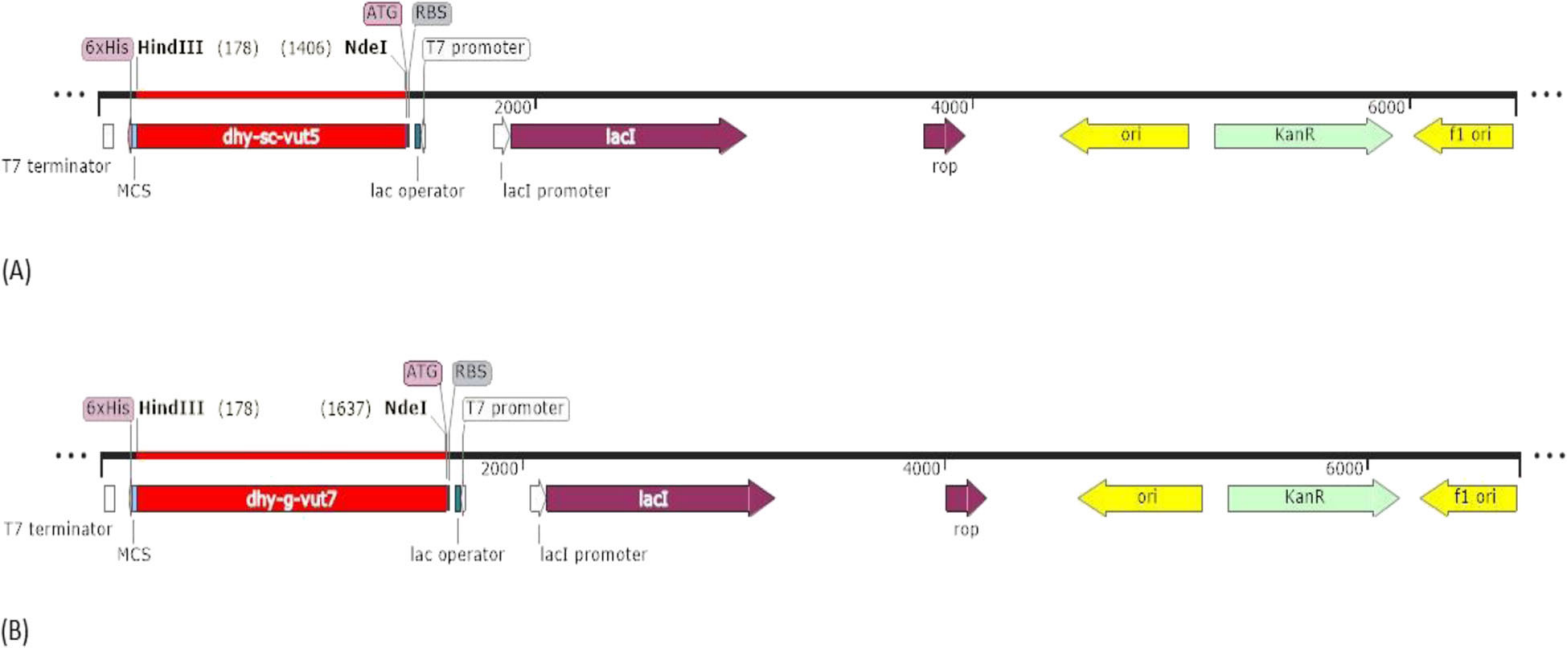
Linearised maps for cloned novel aldehyde dehydrogenase genes in pET30a(+). **a** Linearise map showing features for pET30a(+)_dhy-sc-vut5. **b** Linearised map showing features for pET30(+) _dhy-g-vut7

The two identified ORF sequences designated *dhy-sc-vut5* and *dhy-g-vut7* were codon optimized for codon compatibility in *E. coli* to increase protein expression and His tags were added to the sequence to facilitate for downstream protein purification and detection using the Akta Start Protein Purification System (Merck KGaA, Germany) (Fig. 9a and Fig. 9b). The optimized gene sequences were synthesized by GenScript® USA Inc. The synthesized DNA sequences was cloned into a pET-30a(+) expression vector system (Novagen, USA) with a T7 *lac* promoter according to the pET System manual (Novagen, USA). The DNA sequence was cloned at cloning sites *Nde*I and *Hind*III.

### Transformation of bacterial cells

The preparation of chemically competent *E. coli* BL21 (DE3) pLysS cells was carried out using the Rubidium chloride method [40]. An overnight culture of 0.5 ml was used to inoculate 500 ml LB broth. This was incubated at 37 °C with aeration until the optical density (OD_600_ nm) of 0.48 using the NanoDrop™ One spectrophotometer (Thermo Fisher Scientific, Waltham, USA) was reached. These were decanted into 20 ml tubes and incubated on ice for 15 min followed by centrifugation at 5000×g at 4 °C for 5 min until all cells were pelleted. The supernatant was discarded and 0.4 volumes of Tfbl solution (30 mM Potassium acetate, 100 mM Rubidium Chloride, 10 mM Calcium chloride, 50 mM Manganese chloride and 15% (v/v) glycerol) was used to resuspend the pellet and thereafter kept on ice for 15 min. The cells were pelleted at 5000×g for 5 min at 4 °C and resuspended in 0.04 volumes of TfbII solution pH 6.5 (10 mM MOPS (3-(N-morpholino)propanesulfonic acid), 75 mM Calcium chloride, 10 mM Rubidium chloride and 15% (v/v) glycerol). This solution was kept on ice for 15 min and thereafter 0.5 ml aliquots were placed in 1 ml cryotubes tubes and stored at − 80 °C.

The transformation of the chemically competent *E. coli* BL21 (DE3) pLysS cells with pET-30a(+) expression vector ligated with the gene sequences of interest (*dhy-sc-vut5* and *dhy-g-vut7*) was carried out according to the protocol by for E.cloni® 10G chemically competent cells (Lucigen®). This involved thawing 50 μl of chemically competent *E. coli* BL21 (DE3) pLysS cells on ice and transformation was carried out using 4 μl plasmid vector with insert. The mixture was incubated on ice for 30 min followed by incubation for 45 s at 42 °C in a water bath. The heated solution was placed immediately on ice for 2 min and 960 μl of Recovery medium (E.cloni® 10G, Lucigen). Transformation was also carried out using Rosetta™2 (DE3) *E. coli* cells to check for enhanced expression levels. The culture was allowed to shake at 250 rpm at 37 °C for 1 h and thereafter spread plated onto LB agar plates supplemented with kanamycin (50 μg/ml). This was allowed to grow overnight at 37 °C. A negative control was also setup and used plasmid DNA without insert to transform the host cells. The growth of colonies on the plate indicated the transformed bacteria which were selected for further analysis.

A single transformed colony for each plasmid and insert resulting in pET30(+)_dhy-g-vut7 and pET30(+)_dhy-sc-vut5 were picked and grown in 100 ml LB supplemented with kanamycin (50 μg/ml) overnight at 37 °C with aeration. Glycerol stocks of these were prepared and stored at − 80 °C. In order to carry out quality control and confirm the successful transformation of the *E. coli* host cells, 1.5 ml of the overnight culture was used to isolate plasmid DNA from the pelleted cells using the ZR miniprep plasmid isolation kit (Zymo Research) according to the manufacturer’s instructions. The isolated plasmid DNA was visualized on a 0.8% (w/v) agarose gel stained with ethidium bromide to determine the quality of the plasmid DNA (data not shown, can be supplied if requested). Restriction analysis was also carried out on the plasmid DNA using a double digestion involving the enzymes *XbaI* and *HindIII*. Digestion was allowed to proceed for 3 h at 37 °C and the restriction mixture was separated on a 0.8% (w/v) agarose gel stained with ethidium bromide (data not shown, can be supplied if requested). This was carried out to determine the size of the inserts for each clone and the successful transformation of the *E. coli* host cells.

### Protein induction and expression

The expression of novel His-tagged proteins (DHY-SC-VUT5 and DHY-G-VUT7) was carried out using *E. coli* BL21 (DE3) pLysS cells as the host strain. An overnight seed culture for each of the clones pET30(+)_dhy-g-vut7 and pET30(+)_dhy-sc-vut5 were prepared from glycerol stocks and used to inoculate 200 ml LB broth supplemented with kanamycin (50 μg/ml) with aeration at 37 °C. The cells were grown until they reached an optical density (OD_600_ nm) of 0.5–0.6 using the NanoDrop™ One spectrophotometer (Thermo Fisher Scientific, Waltham, USA) and followed by induction with Isopropyl-β-D-thiogalactoside (IPTG) at a final concentration of 1 mM. The expression was monitored over various time periods at 25 °C with shaking at 250 rpm. A corresponding culture without IPTG was used as the uninduced control. In order to obtain the highest expression levels for each clone, parameters such as growth temperatures (16 °C, 25 °C and 37 °C), the inducer IPTG concentration (0.3–1 mM) final concentration, varied densities of optical density (OD_600_ nm) for induction (0.3–0.9) and the growth period (3 h, 8 h and overnight) were assessed.

Following the various parameters for induction, the bacterial cells were pelleted by centrifugation at 5000 x g for 10 min and protein extraction was carried out using the B-PER® Bacterial Protein Extraction Reagent (Thermo Fisher Scientific, Waltham, USA) according to the manufacturer’s instructions. A test expression protein sample and a solubility protein sample were collected for each expression analysis for induced and uninduced experiments. The uninduced samples represented the negative controls and BSA at 1 μg and 2 μg concentrations were used as a negative control.

### Protein purification and visualization

The expressed protein profile for DHY-SC-VUT5 (pET30(+)_dhy-sc-vut5) and DHY-G-VUT7 (pET30(+)_dhy-g-vut7) were visualised using Sodium dodecyl sulfate– polyacrylamide gel electrophoresis (SDS–PAGE) (15% resolving gel and 5% stacking gel) as described by the Laemmli procedure [41]. Protein samples were prepared by adding Laemmli 2x concentrate sample buffer (Sigma-Aldrich) to the protein supernatant and this was heat denatured at 95 °C in a water bath for 5 min. The proteins were stained with Coomassie blue stain solution (40% ethanol, 0.125% Coomassie® blue, distilled water and 10% acetic acid) and destained with Coomassie blue destain solution (5% ethanol and 7.5% acetic acid). Crude extracts were centrifugation at 18,000 g for 30 min to remove any particulate matter and applied to a nitrilotriacetic acid (Ni-NTA) column (Bio-Rad, Berkeley, CA, USA) according to the manufacturer’s instruction using the Akta Start Protein Purification System (Merck KGaA, Germany). The unbound proteins were washed out with a wash buffer (50 mM KH_2_PO_4_–K_2_HPO_4_, 300 mM NaCl, 40 mM imidazole, pH 8.0). His-tagged fusion proteins were eluted with an elution buffer (50 mM KH_2_PO_4_–K_2_HPO_4_, 300 mM NaCl, 300 mM imidazole, pH 8.0). Fractionation was set to 1.5 ml volumes. This was further dialyzed against 50 mM KH_2_PO_4_–K_2_HPO_4_ (pH 8.0) containing 20% glycerol. Protein concentrations were determined by the Bradford method using bovine serum albumin (BSA, Sigma Aldrich) as a standard [42].

### Western blot analysis

The purified proteins (DHY-SC-VUT5 and DHY-G-VUT7) were visualised using Sodium dodecyl sulfate– polyacrylamide gel electrophoresis (SDS–PAGE) (15% resolving gel and 5% stacking gel) as described by the Laemmli procedure [41]. The iBind™ Western automated system (iBlot 2™ and iBind™) was used to carry out Western blotting according to the manufacturer’s instructions included in the iBlot 2 and iBind Western Starter Kit. Following the blotting, the membranes were blocked with a 1X iBind™ solution. Mouse-anti-His mAb (Thermo Fisher Scientific, Waltham, USA) was used as the primary antibodies for the antibody binding step. Proteins were detected using SuperSignal™ West Pico PLUS Chemiluminescent Substrate (Thermo Fisher Scientific, Waltham, USA) that is an enhanced chemiluminescent (ECL) horseradish peroxidase (HRP) substrate according to manufacturer’s instructions.

### Aldehyde dehydrogenase (ADH) activity assay

The activity of the purified proteins (DHY-SC-VUT5 and DHY-G-VUT7) were carried out according to the protocol by Li et al. [14] for assessing ALDH activity. The standard reaction mixture for the oxidation reaction (200 μl) contained hexanal (Merck, USA) as the substrate, 50 mM Nicotinamide adenine dinucleotide (NAD) in 50 mM Tris-HCl (pH 8.0) and an appropriate amount of enzyme. Varying concentrations of hexanal substrate (25 mM, 50 mM, 100 mM, 150 mM and 200 mM) and enzyme concentrations (0.001 mg/ml, 0.003 mg/ml, 0.007 mg/ml and 0.014 mg/ml) were tested respectively in order to determine the optimal concentrations for each. ALDH activity was assayed spectrophotometrically at 340 nm in a 96 well plate by determining the Nicotinamide adenine dinucleotide phosphate (NADH) produced or oxidized, using the BioTek® Epoch 2 microplate reader (Agilent Technologies, USA). The enzymes were added to the reaction mixture to start the reaction. This was mixed thoroughly and immediately placed in the microplate reader. The absorbance values were recorded at twenty second intervals for 3 min at 30 °C. The enzymatic activity of the purified proteins was measured by the increase in A_340_ due to the formation of NADH. The relative activity for each enzyme was calculated as a percentage by dividing the absorbance range by the highest absorbance value.

### Kinetic studies of novel DHY-SC-VUT5 and DHY-G-VUT7 dehydrogenases

A range of hexanal concentrations (25 mM, 50 mM, 100 mM, 150 mM and 200 mM) were prepared to study the effect of hexanal concentration on the activity of the enzyme, to determine the substrate affinity of the recombinant alcohol dehydrogenase and carry out kinetic studies to obtain the Michaelis-Menten constant (K_m_) and V_max_ using GraphPad Prism for Michaelis-Menten kinetics. The equation of Beer’s Law shown below was used to determine the initial velocity for each substrate concentration at a specific time interval. The wavelength specific absorption co-efficient for NADH (ε) is 6220 mol.L^− 1^ cm^− 1^ and a path length of 0.58 cm was used for a 96 well plate with 200 μl volume.

$$ \mathbf{A} =\boldsymbol{\varepsilon} \boldsymbol{\iota} \boldsymbol{c} $$

**Where:**

**ε** = Substance and wavelength specific absorption coefficient,

***l*** = Length of the light that travels through the sample.

**c =** Sample concentration.

### Statistical analysis

Two-way ANOVA analysis was carried out to determine effect of hexanal concentration and optimal enzyme concentration independently on relative activity. The enzymatic kinetic data was fitted into Michaelis-Menten model using GraphPad Prism Version 8.4.1 (460).

## Supplementary Information

**Additional file 1.**

## Data Availability

All data generated or analysed during this study are included in this published article and its supplementary information files.
