# Supplementary material for: Mining of two novel aldehyde dehydrogenases (DHY-SC-VUT5 and DHY-G-VUT7) from metagenome of hydrocarbon contaminated soils
Source: BMC Biotechnol. 2021 Mar 1;21:18. doi: 10.1186/s12896-021-00677-8 (PMC7923466; doi:10.1186/s12896-021-00677-8)
Supplement: Supplementary file 1 — Additional file 1. [file 12896_2021_677_MOESM1_ESM.docx]

**Mining of Two Novel Aldehyde Dehydrogenases (DHY-SC-VUT5 and DHY-G-VUT7) from Metagenome of Hydrocarbon Contaminated Soils**

Cindy Baburam, Naser Aliye Feto^*^

OMICS Research Group, Department of Biotechnology, Vaal University of Technology, Vanderbijlpark 1911, South Africa.

*Correspondence: [naserf@vut.ac.za/anaser22@yahoo.com](mailto:naserf@vut.ac.za/anaser22@yahoo.com)

**TS 1: Aldehyde** **dehydrogenase DHY-SC-VUT5 sequence data.**

| ***Item*** | **Gene sequence** | **Protein sequence** | **Enzyme** | **Description** | **GenBank Acc.** |
| --- | --- | --- | --- | --- | --- |
| *dhy-sc-vut5* | ATGGCAGTAAAGATTTCAGGAGTCCTGAAAGACGGCACAGGAAAACCGGTACAGAACTGCACCATTCAGCTGAAAGCCAGACGTAACAGCACCACGGTGGTGGTGAACACGGTGGGCTCAGAGAATCCGGATGAAGCCGGGCGTTACAGCATGGATGTGGAGTACGGTCAGTACAGTGTCATCCTGCAGGTTGACGGTTTTCCACCATCGCACGCCGGGACCATCACCGTGTATGAAGATTCACAACCGGGGACGCTGAATGATTTTCTCTGTGCCATGACGGAGGATGATGCCCGGCCGGAGGTGCTGCGTCGTCTTGAACTGATGGTGGAAGAGGTGGCGCGTAACGCGTCCGTGGTGGCACAGAGTACGGCAGACGCGAAGAAATCAGCCGGCGATGCCAGTGCATCAGCTGCTCAGGTCGCGGCCCTTGTGACTGATGCAACGACTCAGCACGCGCCGCCAGCACGTCCGCCGGACAGGCTGCATCGTCAGCTCAGGAAGCGTCCTCCGGCGCAGAAGCGGCATCAGCAAAGGCCACTGAAGCGGAAAAAAGTGCCGCAGCCGCAGAGTCCTCAAAAAACGCGGCGGCCACCAGTGCCGGTGCGGCGAAAACGTCAGAAACGAATGCTGCAGCGTCACAACAATCAGCCGCCACGTCTGCCTCCACCGCGGCCACGAAAGCGTCAGAGGCCGCCACTTCAGCACGAGATGCGGTGGCCTCAAAAGAGGCAGCAAAATCATCAGAAACGAACGCATCATCAAGTGCCGGTCGTGCAGCTTCCTCGGCAACGGCGGCAGAAAATTCTGCCAGGGCGGCAAAAACGTCCGAGACGAATGCCAGGTCATCTGAAACAGCAGCGGAACGGAGCGCCTCTGCCGCGGCAGACGCAAAAACAGCGGCGGCGGGGAGTGCGTCAACGGCATCCACGAAGGCGACAGAGGCTGCGGGAAGTGCGGTATCAGCATCGCAGAGCAAAAGTGCGGCAGAAGCGGCGGCAATACGTGCAGAAAATTCGGCAAAACGTGCAGAAGATATAGCTTCAGCTGTCGCGCTTGAGGATGCGGACACAACGAGAAAGGGGATAGTGCAGCTCAGCAGTGCAACCAACAGCACGTCTGAAACGCTTGCTGCAACGCCAAAGGCGGTTAAGGTGGTAATGGATGAAACGAACAGAAAAGCCCACTGGACAGTCCGGCACTGA | MAVKISGVLKDGTGKPVQNCTIQLKARRNSTTVVVNTVGSENPDEAGRYSMDVEYGQYSVILQVDGFPPSHAGTITVYEDSQPGTLNDFLCAMTEDDARPEVLRRLELMVEEVARNASVVAQSTADAKKSAGDASASAAQVAALVTDATDSARAASTSAGQAASSAQEASSGAEAASAKATEAEKSAAAAESSKNAAATSAGAAKTSETNAAASQQSAATSASTAATKASEAATSARDAVASKEAAKSSETNASSSAGRAASSATAAENSARAAKTSETNARSSETAAERSASAAADAKTAAAGSASTASTKATEAAGSAVSASQSKSAAEAAAIRAENSAKRAEDIASAVALEDADTTRKGIVQLSSATNSTSETLAATPKAVKVVMDETNRKAHWTVRH | Dehydrogenase | Short-chain dehydrogenase exhibited similarity in sequence to that found in *E. coli* | MT606180 |
|  |  |  |  |  |  |
| **TS 2: Aldehyde dehydrogenase DHY-G-VUT7 sequence data.** | | | | | |
| ***Item*** | **Gene sequence** | **Protein sequence** | **Enzyme** | **Description** | **GenBank Acc.** |
| *dhy-g-vut7* | TTGCCCAGCAGGTCCGTGAAACGATGGAGCGCCGTGCAGCCGGTCTTAAACCGCCCGCCTGGGCGGCTGCAGCATTTGAATCCGGGCTGCGACAATCAACAGAGGAGGAGAAGAGTGACAGCAGAGCTGCGTAATCTCCCGCATATTGCCAGCATGGCCTTTAATGAGCCGCTGATGCTTGAACCCGCCTATGCGCGGGTTTTCTTTTGTGCGCTTGCAGGCCAGCTTGGGATCAGCAGCCTGACGGATGCGGTGTCCGGCGACAGCCTGACTGCCCAGGAGGCACTCGCGACGCTGGCATTATCCGGTGATGATGACGGACCACGACAGGCCCGCAGTTATCAGGTCATGAACGGCATCGCCGTGCTGCCGGTGTCCGGCACGCTGGTCAGCCGGACGCGGGCGCTGCAGCCGTACTCGGGGATGACCGGTTACAACGGCATTATCGCCCGTCTGCAACAGGCTGCCAGCGATCCGATGGTGGACGGCATTCTGCTCGATATGGACACGCCCGGCGGGATGGTGGCGGGGGCATTTGACTGCGCTGACATCATCGCCCGTGTGCGTGACATAAAACCGGTATGGGCGCTTGCCAACGACATGAACTGCAGTGCAGGTCAGTTGCTTGCCAGTGCCGCCTCCCGGCGTCTGGTCACGCAGACCGCCCGGACAGGCTCCATCGGCGTCATGATGGCTCACAGTAATTACGGTGCTGCGCTGGAGAAACAGGGTGTGGAAATCACGCTGATTTACAGCGGCAGCCATAAGGTGGATGGCAACCCCTACAGCCATCTTCCGGATGACGTCCGGGAGACACTGCAGTCCCGGATGGACGCAACCCGCCAGATGTTTGCGCAGAAGGTGTCGGCATATACCGGCCTGTCCGTGCAGGTTGTGCTGGATACCGAGGCTGCAGTGTACAGCGGTCAGGAGGCCATTGATGCCGGACTGGCTGATGAACTTGTTAACAGCACCGATGCGATCACCGTCATGCGTGATGCACTGGATGCACGTAAATCCCGTCTCTCAGGAGGGCGAATGACCAAAGAGACTCAATCAACAACTGTTTCAGCCACTGCTTCGCAGGCTGACGTTACTGACGTGGTGCCAGCGACGGAGGGCGAGAACGCCAGCGCGGCGCAGCCGGACGTGAACGCGCAGATCACCGCAGCGGTTGCGGCAGAAAACAGCCGCATTATGGGGATCCTCAACTGTGAGGAGGCTCACGGACGCGAAGAACAGGCACGCGTGCTGGCAGAAACCCCCGGTATGACCGTGAAAACGGCCCGCCGCATTCTGGCCGCAGCACCACAGAGTGCACAGGCGCGCAGTGACACTGCGCTGGATCGTCTGATGCAGGGGGCACCGGCACCGCTGGCTGCAGGTAACCCGGCATCTGATGCCGTTAACGATTTGCTGAACACACCAGTGTAA | MPSRSVKRWSAVQPVLNRPPGRLQHLNPGCDNQQRRRRVTAELRNLPHIASMAFNEPLMLEPAYARVFFCALAGQLGISSLTDAVSGDSLTAQEALATLALSGDDDGPRQARSYQVMNGIAVLPVSGTLVSRTRALQPYSGMTGYNGIIARLQQAASDPMVDGILLDMDTPGGMVAGAFDCADIIARVRDIKPVWALANDMNCSAGQLLASAASRRLVTQTARTGSIGVMMAHSNYGAALEKQGVEITLIYSGSHKVDGNPYSHLPDDVRETLQSRMDATRQMFAQKVSAYTGLSVQVVLDTEAAVYSGQEAIDAGLADELVNSTDAITVMRDALDARKSRLSGGRMTKETQSTTVSATASQADVTDVVPATEGENASAAQPDVNAQITAAVAAENSRIMGILNCEEAHGREEQARVLAETPGMTVKTARRILAAAPQSAQARSDTALDRLMQGAPAPLAAGNPASDAVNDLLNTPV | Dehydrogenase | Glycine dehydrogenase (decarboxylating) exhibited similarity in sequence to that found in *Caulobacter vibrioides* (strain NA1000 / CB15N) (*Caulobacter crescentus*) (EC 1.4.4.2). | MT606181 |

**TS 3: Synthesized genes encoding for aldehyde dehydrogenases after codon optimization as per *E. coli* codon preference.**

| *dhy-sc-vut5* | NdeI**--**ATG**—DHY-SC-VUT5**--His tag--Stop codon‐‐HindIII  CATATGGCGGTGAAAATTAGCGGCGTGCTGAAAGATGGCACCGGCAAACCGGTGCAGAACTGCACCATTCAGCTGAAAGCGCGCCGCAACAGCACCACCGTGGTGGTGAACACCGTGGGCAGCGAAAACCCGGATGAAGCGGGCCGCTATAGCATGGATGTGGAATATGGCCAGTATAGCGTGATTCTGCAGGTGGATGGCTTTCCGCCGAGCCATGCGGGCACCATTACCGTGTATGAAGATAGCCAGCCGGGCACCCTGAACGATTTTCTGTGCGCGATGACCGAAGATGATGCGCGCCCGGAAGTGCTGCGCCGCCTGGAACTGATGGTGGAAGAAGTGGCGCGCAACGCGAGCGTGGTGGCGCAGAGCACCGCGGATGCGAAAAAAAGCGCGGGCGATGCGAGCGCGAGCGCGGCGCAGGTGGCGGCGCTGGTGACCGATGCGACCGATAGCGCGCGCGCGGCGAGCACCAGCGCGGGCCAGGCGGCGAGCAGCGCGCAGGAAGCGAGCAGCGGCGCGGAAGCGGCGAGCGCGAAAGCGACCGAAGCGGAAAAAAGCGCGGCGGCGGCGGAAAGCAGCAAAAACGCGGCGGCGACCAGCGCGGGCGCGGCGAAAACCAGCGAAACCAACGCGGCGGCGAGCCAGCAGAGCGCGGCGACCAGCGCGAGCACCGCGGCGACCAAAGCGAGCGAAGCGGCGACCAGCGCGCGCGATGCGGTGGCGAGCAAAGAAGCGGCGAAAAGCAGCGAAACCAACGCGAGCAGCAGCGCGGGCCGCGCGGCGAGCAGCGCGACCGCGGCGGAAAACAGCGCGCGCGCGGCGAAAACCAGCGAAACCAACGCGCGCAGCAGCGAAACCGCGGCGGAACGCAGCGCGAGCGCGGCGGCGGATGCGAAAACCGCGGCGGCGGGCAGCGCGAGCACCGCGAGCACCAAAGCGACCGAAGCGGCGGGCAGCGCGGTGAGCGCGAGCCAGAGCAAAAGCGCGGCGGAAGCGGCGGCGATTCGCGCGGAAAACAGCGCGAAACGCGCGGAAGATATTGCGAGCGCGGTGGCGCTGGAAGATGCGGATACCACCCGCAAAGGCATTGTGCAGCTGAGCAGCGCGACCAACAGCACCAGCGAAACCCTGGCGGCGACCCCGAAAGCGGTGAAAGTGGTGATGGATGAAACCAACCGCAAAGCGCATTGGACCGTGCGCCATCATCATCATCATCATTAATAAAAGCTT |
| --- | --- |
| *dhy-g-vut7* | NdeI**--**ATG**—DHY-G-VUT7**--His tag--Stop codon‐‐HindIII  CATATGCCGAGCCGCAGCGTGAAACGCTGGAGCGCGGTGCAGCCGGTGCTGAACCGCCCGCCGGGCCGCCTGCAGCATCTGAACCCGGGCTGCGATAACCAGCAGCGCCGCCGCCGCGTGACCGCGGAACTGCGCAACCTGCCGCATATTGCGAGCATGGCGTTTAACGAACCGCTGATGCTGGAACCGGCGTATGCGCGCGTGTTTTTTTGCGCGCTGGCGGGCCAGCTGGGCATTAGCAGCCTGACCGATGCGGTGAGCGGCGATAGCCTGACCGCGCAGGAAGCGCTGGCGACCCTGGCGCTGAGCGGCGATGATGATGGCCCGCGCCAGGCGCGCAGCTATCAGGTGATGAACGGCATTGCGGTGCTGCCGGTGAGCGGCACCCTGGTGAGCCGCACCCGCGCGCTGCAGCCGTATAGCGGCATGACCGGCTATAACGGCATTATTGCGCGCCTGCAGCAGGCGGCGAGCGATCCGATGGTGGATGGCATTCTGCTGGATATGGATACCCCGGGCGGCATGGTGGCGGGCGCGTTTGATTGCGCGGATATTATTGCGCGCGTGCGCGATATTAAACCGGTGTGGGCGCTGGCGAACGATATGAACTGCAGCGCGGGCCAGCTGCTGGCGAGCGCGGCGAGCCGCCGCCTGGTGACCCAGACCGCGCGCACCGGCAGCATTGGCGTGATGATGGCGCATAGCAACTATGGCGCGGCGCTGGAAAAACAGGGCGTGGAAATTACCCTGATTTATAGCGGCAGCCATAAAGTGGATGGCAACCCGTATAGCCATCTGCCGGATGATGTGCGCGAAACCCTGCAGAGCCGCATGGATGCGACCCGCCAGATGTTTGCGCAGAAAGTGAGCGCGTATACCGGCCTGAGCGTGCAGGTGGTGCTGGATACCGAAGCGGCGGTGTATAGCGGCCAGGAAGCGATTGATGCGGGCCTGGCGGATGAACTGGTGAACAGCACCGATGCGATTACCGTGATGCGCGATGCGCTGGATGCGCGCAAAAGCCGCCTGAGCGGCGGCCGCATGACCAAAGAAACCCAGAGCACCACCGTGAGCGCGACCGCGAGCCAGGCGGATGTGACCGATGTGGTGCCGGCGACCGAAGGCGAAAACGCGAGCGCGGCGCAGCCGGATGTGAACGCGCAGATTACCGCGGCGGTGGCGGCGGAAAACAGCCGCATTATGGGCATTCTGAACTGCGAAGAAGCGCATGGCCGCGAAGAACAGGCGCGCGTGCTGGCGGAAACCCCGGGCATGACCGTGAAAACCGCGCGCCGCATTCTGGCGGCGGCGCCGCAGAGCGCGCAGGCGCGCAGCGATACCGCGCTGGATCGCCTGATGCAGGGCGCGCCGGCGCCGCTGGCGGCGGGCAACCCGGCGAGCGATGCGGTGAACGATCTGCTGAACACCCCGGTGCATCATCATCATCATCATTAATAAAAGCTT |

**TS 4: Protein sequences of aldehyde dehydrogenases.**

| *dhy-sc-vut5* | MAVKISGVLKDGTGKPVQNCTIQLKARRNSTTVVVNTVGSENPDEAGRYSMDVEYGQYSVILQVDGFPPSHAGTITVYEDSQPGTLNDFLCAMTEDDARPEVLRRLELMVEEVARNASVVAQSTADAKKSAGDASASAAQVAALVTDATDSARAASTSAGQAASSAQEASSGAEAASAKATEAEKSAAAAESSKNAAATSAGAAKTSETNAAASQQSAATSASTAATKASEAATSARDAVASKEAAKSSETNASSSAGRAASSATAAENSARAAKTSETNARSSETAAERSASAAADAKTAAAGSASTASTKATEAAGSAVSASQSKSAAEAAAIRAENSAKRAEDIASAVALEDADTTRKGIVQLSSATNSTSETLAATPKAVKVVMDETNRKAHWTVRHHHHHH |
| --- | --- |
| *dhy-g-vut7* | MPSRSVKRWSAVQPVLNRPPGRLQHLNPGCDNQQRRRRVTAELRNLPHIASMAFNEPLMLEPAYARVFFCALAGQLGISSLTDAVSGDSLTAQEALATLALSGDDDGPRQARSYQVMNGIAVLPVSGTLVSRTRALQPYSGMTGYNGIIARLQQAASDPMVDGILLDMDTPGGMVAGAFDCADIIARVRDIKPVWALANDMNCSAGQLLASAASRRLVTQTARTGSIGVMMAHSNYGAALEKQGVEITLIYSGSHKVDGNPYSHLPDDVRETLQSRMDATRQMFAQKVSAYTGLSVQVVLDTEAAVYSGQEAIDAGLADELVNSTDAITVMRDALDARKSRLSGGRMTKETQSTTVSATASQADVTDVVPATEGENASAAQPDVNAQITAAVAAENSRIMGILNCEEAHGREEQARVLAETPGMTVKTARRILAAAPQSAQARSDTALDRLMQGAPAPLAAGNPASDAVNDLLNTPVHHHHHH |

**TS 5: Absorbance values for varying aldehyde dehydrogenase DHY-SC-VUT5 concentration.**

| **Time(sec)** | **0.001 mg/ml** | | **0.003 mg/ml** | | **0.007 mg/ml** | | **0.014 mg/ml** | |
| --- | --- | --- | --- | --- | --- | --- | --- | --- |
|  | **Read 1** | **Read 2** | **Read 1** | **Read 2** | **Read 1** | **Read 2** | **Read 1** | **Read 2** |
| 0 | 0.000 | 0.000 | 0.000 | 0.000 | 0.000 | 0.000 | 0.000 | 0.000 |
| 20 | 0.475 | 0.424 | 0.452 | 0.527 | 0.486 | 0.465 | 0.374 | 0.37 |
| 40 | 0.477 | 0.416 | 0.425 | 0.558 | 0.48 | 0.465 | 0.387 | 0.376 |
| 60 | 0.476 | 0.444 | 0.427 | 0.547 | 0.479 | 0.464 | 0.388 | 0.376 |
| 80 | 0.481 | 0.429 | 0.425 | 0.555 | 0.478 | 0.463 | 0.387 | 0.374 |
| 100 | 0.478 | 0.43 | 0.442 | 0.562 | 0.476 | 0.462 | 0.387 | 0.374 |
| 120 | 0.473 | 0.433 | 0.456 | 0.558 | 0.475 | 0.462 | 0.385 | 0.373 |
| 140 | 0.484 | 0.43 | 0.45 | 0.553 | 0.474 | 0.461 | 0.384 | 0.371 |
| 160 | 0.475 | 0.431 | 0.441 | 0.552 | 0.473 | 0.461 | 0.383 | 0.371 |
| 180 | 0.471 | 0.41 | 0.433 | 0.552 | 0.471 | 0.461 | 0.381 | 0.37 |
| 200 | 0.469 | 0.419 | 0.441 | 0.554 | 0.47 | 0.464 | 0.38 | 0.369 |
| 220 | 0.469 | 0.44 | 0.442 | 0.553 | 0.469 | 0.464 | 0.379 | 0.368 |
| 240 | 0.47 | 0.434 | 0.452 | 0.551 | 0.468 | 0.464 | 0.378 | 0.368 |
| 260 | 0.472 | 0.42 | 0.44 | 0.553 | 0.467 | 0.466 | 0.377 | 0.368 |
| 280 | 0.471 | 0.429 | 0.441 | 0.552 | 0.466 | 0.466 | 0.376 | 0.366 |
| 300 | 0.482 | 0.41 | 0.436 | 0.552 | 0.465 | 0.466 | 0.376 | 0.366 |

**TS 6: Absorbance values for varying aldehyde dehydrogenase DHY-G-VUT7 concentration.**

| **Time(sec)** | **0.001 mg/ml** | | **0.003 mg/ml** | | **0.007 mg/ml** | | **0.014 mg/ml** | |
| --- | --- | --- | --- | --- | --- | --- | --- | --- |
|  | **Read 1** | **Read 2** | **Read 1** | **Read 2** | **Read 1** | **Read 2** | **Read 1** | **Read 2** |
| 0 | 0.000 | 0.000 | 0.000 | 0.000 | 0.000 | 0.000 | 0.000 | 0.000 |
| 20 | 0.366 | 0.35 | 0.53 | 0.333 | 0.483 | 0.369 | 0.374 | 0.355 |
| 40 | 0.36 | 0.354 | 0.531 | 0.332 | 0.494 | 0.373 | 0.371 | 0.353 |
| 60 | 0.363 | 0.351 | 0.526 | 0.332 | 0.495 | 0.371 | 0.369 | 0.352 |
| 80 | 0.362 | 0.348 | 0.522 | 0.331 | 0.492 | 0.369 | 0.367 | 0.355 |
| 100 | 0.36 | 0.348 | 0.523 | 0.329 | 0.492 | 0.365 | 0.365 | 0.35 |
| 120 | 0.357 | 0.348 | 0.524 | 0.328 | 0.488 | 0.362 | 0.365 | 0.347 |
| 140 | 0.354 | 0.347 | 0.531 | 0.329 | 0.481 | 0.362 | 0.363 | 0.346 |
| 160 | 0.357 | 0.347 | 0.527 | 0.328 | 0.485 | 0.36 | 0.361 | 0.343 |
| 180 | 0.357 | 0.347 | 0.529 | 0.327 | 0.483 | 0.356 | 0.36 | 0.343 |
| 200 | 0.356 | 0.351 | 0.524 | 0.325 | 0.482 | 0.356 | 0.359 | 0.342 |
| 220 | 0.352 | 0.354 | 0.519 | 0.324 | 0.482 | 0.356 | 0.358 | 0.341 |
| 240 | 0.354 | 0.355 | 0.524 | 0.325 | 0.482 | 0.354 | 0.357 | 0.346 |
| 260 | 0.352 | 0.357 | 0.515 | 0.323 | 0.482 | 0.353 | 0.356 | 0.339 |
| 280 | 0.351 | 0.357 | 0.523 | 0.323 | 0.479 | 0.353 | 0.356 | 0.339 |
| 300 | 0.35 | 0.357 | 0.513 | 0.322 | 0.476 | 0.352 | 0.355 | 0.343 |

**TS 7: Absorbance values for activity of aldehyde dehydrogenase DHY-SC-VUT5 at varying hexanal concentration.**

| **Time(sec)** | **25mM** | | **50 mM** | | **100 mM** | | **150 mM** | | **200 mM** | |
| --- | --- | --- | --- | --- | --- | --- | --- | --- | --- | --- |
|  | **Read1** | **Read2** | **Read1** | **Read2** | **Read1** | **Read2** | **Read1** | **Read2** | **Read1** | **Read2** |
| 0 | 0.000 | 0.000 | 0.000 | 0.000 | 0.000 | 0.000 | 0.000 | 0.000 | 0.000 | 0.000 |
| 20 | 0.344 | 0.387 | 0.394 | 0.35 | 0.378 | 0.367 | 0.368 | 0.423 | 0.368 | 0.415 |
| 40 | 0.346 | 0.385 | 0.396 | 0.344 | 0.376 | 0.363 | 0.369 | 0.422 | 0.366 | 0.424 |
| 60 | 0.347 | 0.386 | 0.395 | 0.345 | 0.376 | 0.361 | 0.369 | 0.421 | 0.364 | 0.424 |
| 80 | 0.346 | 0.389 | 0.395 | 0.351 | 0.375 | 0.361 | 0.369 | 0.42 | 0.365 | 0.419 |
| 100 | 0.345 | 0.39 | 0.394 | 0.346 | 0.374 | 0.361 | 0.369 | 0.419 | 0.364 | 0.417 |
| 120 | 0.343 | 0.391 | 0.394 | 0.347 | 0.373 | 0.362 | 0.368 | 0.418 | 0.365 | 0.419 |
| 140 | 0.347 | 0.392 | 0.394 | 0.347 | 0.373 | 0.362 | 0.368 | 0.418 | 0.365 | 0.42 |
| 160 | 0.343 | 0.394 | 0.393 | 0.345 | 0.372 | 0.363 | 0.368 | 0.417 | 0.366 | 0.418 |
| 180 | 0.344 | 0.394 | 0.393 | 0.347 | 0.372 | 0.363 | 0.367 | 0.417 | 0.366 | 0.417 |
| 200 | 0.345 | 0.395 | 0.393 | 0.35 | 0.371 | 0.363 | 0.368 | 0.417 | 0.366 | 0.42 |
| 220 | 0.344 | 0.395 | 0.392 | 0.349 | 0.371 | 0.363 | 0.367 | 0.416 | 0.365 | 0.417 |
| 240 | 0.346 | 0.395 | 0.392 | 0.349 | 0.371 | 0.363 | 0.367 | 0.416 | 0.366 | 0.421 |
| 260 | 0.347 | 0.395 | 0.392 | 0.351 | 0.371 | 0.364 | 0.367 | 0.416 | 0.365 | 0.417 |
| 280 | 0.346 | 0.395 | 0.392 | 0.352 | 0.37 | 0.364 | 0.367 | 0.415 | 0.365 | 0.415 |
| 300 | 0.346 | 0.396 | 0.391 | 0.354 | 0.371 | 0.364 | 0.367 | 0.414 | 0.365 | 0.416 |

**TS 8: Absorbance values for activity of aldehyde dehydrogenase DHY-G-VUT7 at varying hexanal concentration.**

| **Time(sec)** | **25mM** | | **50 mM** | | **100 mM** | | **150 mM** | | **200 mM** | |
| --- | --- | --- | --- | --- | --- | --- | --- | --- | --- | --- |
|  | **Read1** | **Read2** | **Read1** | **Read2** | **Read1** | **Read2** | **Read1** | **Read2** | **Read1** | **Read2** |
| 0 | 0.000 | 0.000 | 0.000 | 0.000 | 0.000 | 0.000 | 0.000 | 0.000 | 0.000 | 0.000 |
| 20 | 0.344 | 0.355 | 0.365 | 0.369 | 0.347 | 0.334 | 0.362 | 0.349 | 0.349 | 0.372 |
| 40 | 0.348 | 0.361 | 0.363 | 0.365 | 0.355 | 0.337 | 0.365 | 0.363 | 0.354 | 0.377 |
| 60 | 0.352 | 0.362 | 0.366 | 0.364 | 0.357 | 0.34 | 0.369 | 0.368 | 0.363 | 0.379 |
| 80 | 0.353 | 0.362 | 0.368 | 0.364 | 0.357 | 0.34 | 0.369 | 0.373 | 0.367 | 0.378 |
| 100 | 0.354 | 0.363 | 0.372 | 0.365 | 0.356 | 0.338 | 0.368 | 0.373 | 0.369 | 0.378 |
| 120 | 0.354 | 0.362 | 0.371 | 0.364 | 0.356 | 0.339 | 0.368 | 0.378 | 0.372 | 0.38 |
| 140 | 0.353 | 0.363 | 0.371 | 0.364 | 0.356 | 0.339 | 0.367 | 0.38 | 0.372 | 0.376 |
| 160 | 0.353 | 0.362 | 0.372 | 0.362 | 0.356 | 0.339 | 0.367 | 0.38 | 0.374 | 0.379 |
| 180 | 0.352 | 0.362 | 0.371 | 0.363 | 0.356 | 0.339 | 0.366 | 0.381 | 0.374 | 0.379 |
| 200 | 0.352 | 0.361 | 0.37 | 0.362 | 0.358 | 0.338 | 0.367 | 0.381 | 0.374 | 0.375 |
| 220 | 0.353 | 0.365 | 0.37 | 0.361 | 0.357 | 0.337 | 0.367 | 0.38 | 0.374 | 0.372 |
| 240 | 0.352 | 0.364 | 0.371 | 0.362 | 0.357 | 0.338 | 0.367 | 0.383 | 0.373 | 0.378 |
| 260 | 0.353 | 0.364 | 0.371 | 0.361 | 0.357 | 0.339 | 0.367 | 0.383 | 0.373 | 0.377 |
| 280 | 0.353 | 0.364 | 0.369 | 0.362 | 0.357 | 0.338 | 0.367 | 0.383 | 0.374 | 0.378 |
| 300 | 0.352 | 0.365 | 0.37 | 0.361 | 0.357 | 0.339 | 0.367 | 0.381 | 0.373 | 0.374 |
